# Reduced axonal transport in Parkinson's disease cybrid neurites is restored by light therapy

**DOI:** 10.1186/1750-1326-4-26

**Published:** 2009-06-17

**Authors:** Patricia A Trimmer, Kathleen M Schwartz, M Kathleen Borland, Luis De Taboada, Jackson Streeter, Uri Oron

**Affiliations:** 1 University of Virginia, Morris K Udall Parkinson's Research Center of Excellence and Department of Neurology, Charlottesville, Virginia, USA; 2 PhotoThera, Inc, Carlsbad, California, USA; 3 Tel-Aviv University, Department of Zoology, Tel-Aviv, Israel

## Abstract

**Background:**

It has been hypothesized that reduced axonal transport contributes to the degeneration of neuronal processes in Parkinson's disease (PD). Mitochondria supply the adenosine triphosphate (ATP) needed to support axonal transport and contribute to many other cellular functions essential for the survival of neuronal cells. Furthermore, mitochondria in PD tissues are metabolically and functionally compromised. To address this hypothesis, we measured the velocity of mitochondrial movement in human transmitochondrial cybrid "cytoplasmic hybrid" neuronal cells bearing mitochondrial DNA from patients with sporadic PD and disease-free age-matched volunteer controls (CNT). The absorption of low level, near-infrared laser light by components of the mitochondrial electron transport chain (mtETC) enhances mitochondrial metabolism, stimulates oxidative phosphorylation and improves redox capacity. PD and CNT cybrid neuronal cells were exposed to near-infrared laser light to determine if the velocity of mitochondrial movement can be restored by low level light therapy (LLLT). Axonal transport of labeled mitochondria was documented by time lapse microscopy in dopaminergic PD and CNT cybrid neuronal cells before and after illumination with an 810 nm diode laser (50 mW/cm^2^) for 40 seconds. Oxygen utilization and assembly of mtETC complexes were also determined.

**Results:**

The velocity of mitochondrial movement in PD cybrid neuronal cells (0.175 +/- 0.005 SEM) was significantly reduced (p < 0.02) compared to mitochondrial movement in disease free CNT cybrid neuronal cells (0.232 +/- 0.017 SEM). For two hours after LLLT, the average velocity of mitochondrial movement in PD cybrid neurites was significantly (p < 0.003) increased (to 0.224 +/- 0.02 SEM) and restored to levels comparable to CNT. Mitochondrial movement in CNT cybrid neurites was unaltered by LLLT (0.232 +/- 0.017 SEM). Assembly of complexes in the mtETC was reduced and oxygen utilization was altered in PD cybrid neuronal cells. PD cybrid neuronal cell lines with the most dysfunctional mtETC assembly and oxygen utilization profiles were least responsive to LLLT.

**Conclusion:**

The results from this study support our proposal that axonal transport is reduced in sporadic PD and that a single, brief treatment with near-infrared light can restore axonal transport to control levels. These results are the first demonstration that LLLT can increase axonal transport in model human dopaminergic neuronal cells and they suggest that LLLT could be developed as a novel treatment to improve neuronal function in patients with PD.

## Background

Sporadic Parkinson's disease (PD) is a disabling, progressive neurodegenerative disease. The neuropathological characterization of PD includes not only the loss of dopaminergic neurons in the substantia nigra and other brainstem nuclei but also the presence of cytoplasmic inclusions that form Lewy bodies and Lewy neurites in surviving neurons. [1,2] Degeneration of nigral neurons begins at synaptic terminals and progresses retrograde to cell bodies before the onset of symptoms. [3] At symptom onset, 60–80% of the striatal dopaminergic terminals have already been lost. Dopaminergic nerve terminals continue to be lost at a rate of 10–12% a year. [4]

Neurons prone to degeneration in PD have axons that are long and poorly myelinated and provide a massive innervation to the striatum (150,000 presynaptic terminals per neuron). [5] Neurons have a high metabolic rate and require a large, uninterrupted supply of ATP. [6] Microtubule-based anterograde axonal transport is ATP-driven and responsible for the delivery of mitochondria and other cellular constituents to distal sites in neurons. Compromised retrograde axonal transport deprives the cell body of vital trophic factors and deprives axons and dendrites of synaptic vesicles, lysosomes and mitochondria. [5,7]

Reduced or compromised axonal transport could underlie the progressive, relentless loss of dopaminergic nerve terminals in sporadic PD. Axonal transport is one aspect of mitochondrial dynamics that is critical for the distribution of functional mitochondria to distal synaptic terminals. Movement, fission, and fusion of mitochondria are functions that are also essential for neuronal survival. [8] Studies at the ultrastructural level in human PD caudate and striatum detected a disruption in the distribution of both microtubules and organelles such as mitochondria. [9] Neurotoxins that are used to model PD pathogenesis (1-methyl-4-phenylpyridinium (MPP+) and rotenone) compromise mitochondrial function by inhibiting complex I of the mtETC. These toxins also have microtubule depolymerizing activities that alter axonal transport. [2,10-14] Complex I of the mtETC is structurally and functionally defective in brain, platelets and other tissues in PD patients. [15,16] Since mitochondrial DNA (mtDNA) encodes components of the mtETC, it is not surprising that mtDNA from PD patients contains increased levels of mutations and deletions. [15,17-19]

It is now known that genes linked to familial PD (α-synuclein, parkin and PINK1) play important roles in mitochondrial transport dynamics and function. [8,20] Pathogenic mutation, hyper-phosphorylation or over-expression of α-synuclein is associated with reduced axonal transport. [21] Liang et al [13] and Feng [22] suggested that the consequences of both genetic and environmental risk factors for familial and sporadic PD disrupt microtubules and axonal transport, leading to the death of dopaminergic neurons.

In an effort to explore the contribution of mitochondria to features of PD pathogenesis such as reduced axonal transport, we have focused our studies on "cytoplasmic hybrid" cybrid cell lines. Cybrid cell lines were created by the fusion of donated platelets from PD or CNT volunteers with human mtDNA-free (Rho0) SH-SY5Y human neuroblastoma or NT2 teratocarcinoma cells. [23,24] Cybrid cell lines have been used to explore the role of mitochondrial dysfunction in PD. [24-28] Using this model of PD pathogenesis, the contribution of mitochondria can be selectively studied and the variability due to nuclear genes and environmental factors can be controlled. [29]

Changes in PD cybrid cell lines correlate with changes seen in PD brain. [29] PD cybrid lines exhibit- A) decreased complex I activity,[26,28] B) increased production of reactive oxygen species (ROS),[26,28,30] C) increased levels of antioxidant enzymes,[26,31] D) increased numbers of morphologically abnormal mitochondria,[32] E) mtDNA with deletions (personal communication, Dr. Bradley Miller), and F) fibrillar and vesicular inclusions called cybrid Lewy bodies that replicate the essential antigenic and structural features of Lewy bodies in PD brain. [27]

Non-dividing, process-bearing, dopaminergic neuronal cells were generated from undifferentiated CNT and PD cybrids using a low concentration of staurosporine. [2,33] These PD and CNT cybrid neuronal cells contain neuronal cytoskeletal proteins (neuron-specific beta tubulin, tau, and microtubule associated protein 2) and dopaminergic markers (tyrosine hydroxylase, dopamine transporter, the dopamine D2 receptor and the vesicular monoamine transporter). [2] Our results show that the velocity of mitochondrial movement is significantly reduced in the processes of PD cybrid neuronal cells compared to CNT. Reduced movement of mitochondria in human PD cybrid neuronal cells supports the proposition that reduced axonal transport plays a role in PD progression.

In an effort to normalize this reduction in mitochondrial movement, we exposed PD and CNT cybrid neuronal cells to a low level near-infrared light therapy. In animal studies, low level near-infrared light therapy (LLLT) improved the outcome in retinal damage due to methanol or rotenone toxicity, [33-35] traumatic brain injury,[36] or spinal cord injury,[37] and induced analgesia in dorsal root ganglion neurons. [38] LLLT has improved the survival and function of rat neuronal cells exposed to MPP+ [39] or rotenone. [35] In the human brain, transcranial laser therapy (delivery of near-infrared laser light through the scalp and skull) has been used to successfully treat complex neurological conditions such as ischemic stroke. [40] Research supports the idea that near-infrared laser (750–1400 nm) light is absorbed and stimulates components in the mtETC, increases redox capacity, activates cytochrome c oxidase (complex IV), increases ATP production in dysfunctional cells and initiates long-term changes in neuronal function in human and animal studies. [37,40-43] Our results show that a single treatment with 810 nm laser light was sufficient to restore the velocity of mitochondrial movement to control levels in PD cybrid neuronal cells.

## Methods

### Patient population

The demographics for PD and CNT volunteers were described previously. [27] The Hoehn and Yahr[44] score for all PD volunteers was stage 2. The mean age for the PD group was 72.14 +/- 4.18SD and 64.4 +/- 5.68SD for the CNT group.

### Cybrid growth and differentiation

PD and CNT cybrid cell lines were created by fusing platelets from sporadic PD or disease-free CNT individuals with mtDNA-free Rho0 cells created in SH-SY5Y neuroblastoma cells. [45] Blood samples were collected under an IRB-approved protocol, and details of cybrid creation were described previously. [27] PD and CNT cybrid, SH-SY5Y and Rho0 cell lines were grown in T75 tissue culture flasks (Greiner, PGC Scientific, Gaithersburg, MD) in a culture medium (CM) consisting of Dulbecco's modified Eagle's medium with high glucose (DMEM), 10% characterized fetal bovine serum (FBS, Hyclone, Logan, Utah), 100 μg/ml pyruvate, 50 μg/ml uridine, antibiotic-antimycotic, 100 Units/ml penicillin G, 100 μg/ml streptomycin, 0.25 μg/ml and amphotericin β (Invitrogen, Carlsbad, California, USA). [27]

### Cybrid neuron differentiation

Glass bottomed 35 mm dishes (MatTek Corp., Ashland, MA) were washed with sterile H_2_O then treated with sterile 1 N HCl for 30 minutes, washed three times with sterile H_2_O and stored at room temperature (RT). Coverslip dishes were coated for 40 min.-overnight with 1.5 mg/ml poly-l-lysine (PL, Sigma-Aldrich, St. Louis, Missouri, USA) dissolved in sterile RT H_2_O. Dishes were rinsed twice with sterile RT H_2_O before adding the cell suspension.

Proliferating cybrid cells were harvested from flasks with 0.05% trypsin (Invitrogen, Carlsbad, CA) in phosphate buffered saline (PBS, Sigma-Aldrich, St. Louis, Missouri, USA) for 5 minutes at 37°C. Trypsin activity was stopped by an equal volume of CM. Cells were then centrifuged at 200 g for 5 minutes and re-suspended in CM. 40,000 cells in 2 ml CM were added to each 35 mM dish. After 18–24 hours, CM was removed and the differentiation media (DM, 500 ml of Neurobasal with 10 ml B27 supplements [Invitrogen, Carlsbad, CA] and 0.5 mM glutamine, pyruvate, uridine and antibiotic-antimycotic as described above). A 25 μM stock solution of staurosporine (ST, Sigma-Aldrich Corp, St Louis, Missouri, USA) was made in dimethyl sulfoxide, frozen at -80°C. Unused aliquots were discarded after 60 days. ST (4 nM-8 nM) dilutions were made fresh in DM and replaced every 2–3 days. Differentiation was completed on day 12.

### Immunocytochemistry

The MitoProfile^® ^OXPHOS/PDH immunocytochemistry kit (MitoSciences, Eugene, Oregon, USA) was used to detect defective assembly of ETC complexes I-V. The manufacturer's suggested protocol was modified for coverslip dishes. Vectashield mounting medium (Vector Labs, Burlingame, California, USA) was added to coverslip dish after staining. Images of stained cybrid neuronal cells were created using an Olympus Fluoview laser scanning confocal microscope system (Center Valley, Pennsylvania, USA). SYTO 61 (Invitrogen) at a concentration of 5 mM (30 min) was used to stain nucleic acids in the cell nucleus and cytoplasm.

### High Resolution Oxygen Respirometry

A two chamber oxygraph O2k (Oroboros, Innsbruck, Austria) was used to measure respiration rates of dissociated CNT, PD and laser-treated CNT and PD cybrid neuronal cells according to manufacturers directions. After 12 days in DM, PD and CNT cybrid neuronal cells were harvested by rinsing with PBS. The cells (4.5 × 10^6 ^cells/ml resuspended in phenol-free DMEM and separated into two sterile 35 mm culture dishes) were kept on ice. In this blinded study, one dish received LLLT (see below) and the other dish remained untreated for each cybrid line studied. After the oxygraph was calibrated according to manufacturer's instructions, 2 ml of LLLT or untreated cybrid neuronal cells were added to each chamber. The chambers were closed and cells were equilibrated (37°C). After the basal rate of respiration was established, complex V respiration was inhibited by oligomycin (4 ug/ml final concentration). Respiration was uncoupled by carbonyl cyanide 4-(trifluoromethoxy)phenylhydrazone (FCCP at a final concentration of ~0.325 uM, Sigma St Louis, MO). Complex 1 was inhibited by adding rotenone at a final concentration of 1.0 μM.

### Axonal transport

To measure mitochondrial movement, cybrid neuronal cells were incubated with 50 nM MitoTracker CMXRos (MTRed; Invitrogen, Carlsbad, California, USA) for 10 min at 37°C. [46] Time-lapse recordings were made using an Olympus IX70 microscope equipped with epifluorescence and Nomarski optics, a Lambda 10-2 filter wheel, a Photometrics CoolSnap HQ progressive scan CCD camera and a heater/controller to maintain cybrid cells at 37°C during image collection (World Precision Instruments, Inc, Sarasota, Florida, USA). Collection of image stacks, and velocity measurements were made using MetaMorph Imaging System (Molecular Devices, Downingtown, Pennsylvania, USA). For standard recordings, images were collected every 3 seconds for 2 min. Unlike other studies, the velocity of all mitochondria in each cell process was tracked by hand whether they moved or not. The average velocity for individual mitochondria was calculated using intervals where movement occurred. [46] Neutral density filters are used to reduce illumination from the mercury lamp and minimize phototoxicity. [46]

### LLLT with near-infrared 810 nm laser

Differentiated neuronal cells from cultures of six PD and five CNT cybrid lines as well as SH-SY5Y were labeled with MtRed in phenol-free DM and 10–15 movies (1–3 neurites per movie, 8–50 mitochondria per process) were collected per dish. A second dish from each line was switched to phenol-free DM and uniformly illuminated at 50 mW/cm^2 ^(measured at the dish surface) with a continuous wave 810nm diode laser for 40 seconds (Acculaser, PhotoThera, Inc, Carlsbad, California, USA). The power output of the laser was measured before each LLLT experiment with an Ohir Optronics power meter (Jerusalem, Israel) to ensure consistent exposure. After LLLT, neuronal cells from six PD, five CNT and SH-SY5Y lines were stained with MTRed as described above. Movies (n = 10–15 movies per experimental condition, 2–3 processes/movie, 8–50 mitochondria per process) were collected at 5 to 10 minute intervals for at least 2 hours for each dish (n = 2–3 experiments +/- LLLT per cybrid line). T-tests assuming equal or unequal variance were used as needed to analyze the data.

## Results

### Mitochondrial movement in differentiated PD and CNT cybrid neurites

**Figure 1:**
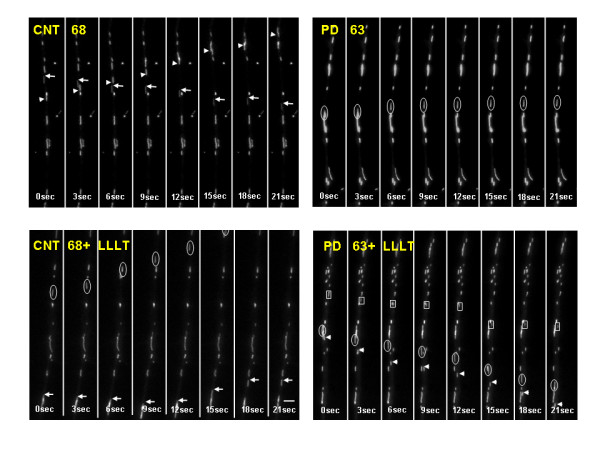
**These montages illustrate the movements of MTRed-labeled mitochondria in the processes of CNT68, CNT68-90 minutes after LLLT, PD63 and PD63-85 minutes after LLLT**. Moving mitochondria in each montage are indicated by arrowheads, arrows, circles and boxes. The images were taken every 3 seconds. Original movies are shown as additional files. Bar = 1 μm.

**Figure 2:**
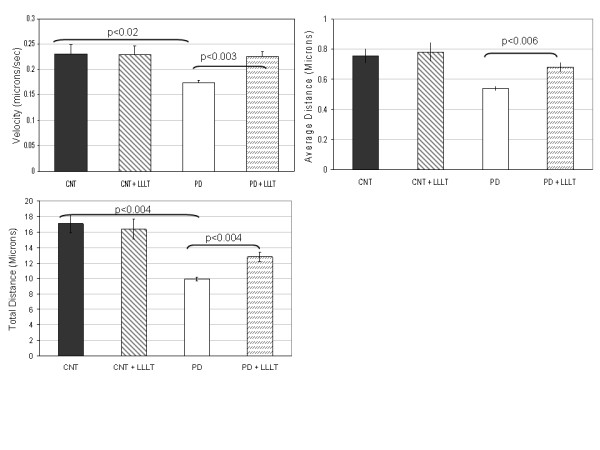
**This figure contains three graphs.** Graph 2A depicts the average velocity of mitochondrial movement in the processes of CNT (n = 5), CNT+LLLT (n = 5), PD (n = 6) and PD+LLLT (n = 6) cybrid neuronal cell lines. Using a two-tailed t-test assuming unequal variance, mitochondrial movement in PD cybrid neurites was significantly reduced compared to CNT. Mitochondrial movement in PD+LLLT cybrid neurites was significantly increased compared to PD. Mitochondrial movment in CNT cybrid neurites was not significantly different from CNT+LLLT. Differences in culture conditions, cell type, mitochondrial labeling method and method of velocity calculation make it difficult to compare mitochondrial velocities among published papers. Graph 2B depicts the average distance traveled by mitochondria during the recording period. The average distance traveled by mitochondria (calculated by averaging the distance traveled for each three second interval irrespective of direction of travel) in PD+LLLT was significantly increased above PD. Graph 2C depicts the total distance traveled (calculated by summing the distance traveled in each three second interval irrespective of direction of travel) during the recording period by mitochondria in each category. The total distance traveled by PD mitochondria was significantly reduced compared to CNT. The total distance traveled by PD+LLLT mitochondria were significantly increased compared to PD.

The processes selected for study in all conditions were comparable in length and diameter (Figure 1). The range of mitochondrial lengths in PD, CNT and LLLT-treated cybrids was not qualitatively different (Figure 1). Mitochondrial movement in PD, CNT and LLLT and untreated cybrid neurites was bidirectional but primarily anterograde and saltatory (see Additional file 1, Additional file 2, Additional file 3, Additional file 4). The number of mitochondria moving retrograde in any process was minimal. Consequently, velocity was measured irrespective of direction. Average velocities for individual mitochondria less than or equal to 0.075 μm/second were categorized as stationary. In CNT cybrids, 14% of the mitochondria were stationary during the recording period. The number of stationary mitochondria in PD cybrid neurites was elevated compared to CNT cybrid neurites (29%, see Table 1). As shown in Table 1 and Figure 2, the average velocity of mitochondrial movement in PD cybrid neurites (0.175 +/- 0.005SEM) was significantly less than the velocity in CNT cybrid neurites (0.232 +/- 0.017SEM). The total amount of time spent moving and the average distance traveled by mitochondria in PD cybrid neurites was reduced but not significantly different from CNT (Table 1, Figure 2) however, the total distance traveled by mitochondria in PD cybrid neurites was significantly reduced compared to CNT cybrid mitochondria (Figure 2).

**Table 1:** Data from studies of mitochondrial movement in LLLT and untreated PD and CNT cybrid neurons

	**PD** (n = 6)	**SEM**	**PD vs PD+LLLT** **p value**	**CNT** (n = 5)	**SEM**	**PD vs CNT** **p value**
Velocity (*μ*m/sec)	0.175	0.005	0.001	0.232	0.017	0.02
Percent not moving (v<0.075)	29	3.68	0.01	14	2.42	0.01
Average distance traveled	0.54	0.016	0.006	0.757	0.048	NS
Total distance traveled	9.92	0.261	0.004	17.09	1.18	0.004
Time spent moving (out of 120 sec)	45	0.358	NS	61	6.89	NS
Number of neurites/cybrid	24–53			26–44		

						

	**PD + LLLT** (n = 6)	**SEM**		**CNT + LLLT** (n = 5)	**SEM**	**CNT vs CNT+LLLT** **p value**

LLLT-50 mW/cm^2 ^for 40 sec (2J/cm^2^)						
Velocity (*μ*m/sec)	0.224	0.02		0.226	0.017	NS
Percent not moving (v<0.075)	12	2.15		16	2.15	NS
Average distance traveled	0.682	0.033		0.782	0.065	NS
Total distance traveled	12.78	0.616		16.34	1.27	NS
Time spent moving (out of 120 sec)	48	3.94		63	6.92	NS
Number of neurites/cybrid	25–51			28–58		

Significance was determined using a two-tailed t test assuming unequal variance.

### Effects of LLLT on mitochondrial movement in differentiated PD and CNT cybrid neurites

For at least 2 hours after exposure to LLLT (Table 1 and Figure 2), the velocity of mitochondrial movement in PD cybrid neurites was significantly increased and restored to CNT levels. This change in velocity was achieved by reducing the number of stationary mitochondria and increasing the average and total distance traveled (Table 1 and Figure 2). Further studies are needed to determine if more individual stationary mitochondria initiate movement after LLLT or if mitochondria already in motion exhibit increased velocity. The total time PD mitochondria spent moving after LLLT was not significantly different from CNT (Table 1). Of the six PD cybrid lines studied, the response of two lines (PD60, PD61) to LLLT was consistently increased but did not achieve significance in three separate studies. Mitochondrial movement in the five CNT cybrid lines also did not change significantly in response to LLLT (Table 1 and Figure 2).

### mtETC assembly in PD, CNT and LLLT-treated cybrid neuronal cells

**Figure 3:**
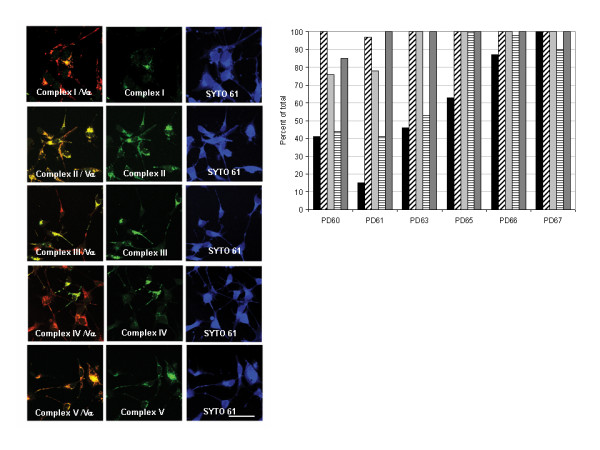
**The montage in 3A shows PD61 cybrid neuronal cells stained with the MitoProfile^® ^OXPHOS/PDH immunocytochemistry kit (MitoSciences)**. Specific antibodies (FITC secondary antibody, green) to Complex I-NDUFB4 (A-C), Complex II-30 kDa (D-F), Complex III-Core 2 protein (G-I), Complex IV-subunit I (J-L), and Complex V-OSCP (M-O) were combined with antibodies to a control subunit (CVα, Texas red secondary, red) to double stain mitochondria in cybrid neuronal cells (see methods). The cell bodies were stained with SYTO 61 (blue). Cybrid neuronal cells in the first column that exhibit double staining are orange or yellow and have intact mETC assembly while cybrid neuronal cells that lack intact assembly of complexs in the mETC are red. Bar = 20 μm. The graph in 3B depicts counts of PD cybrid neuronal cells that contained fully assembled mtETC complexes. In this graph Complex I is black, Complex II has diagonal stripes, Complex III is light gray, Complex IV has horizontal stripes, and Complex V is dark gray. The CNT cybrid neuronal cells exhibited complete assembly of all five complexes (data not shown).

To better understand the role that mitochondrial dysfunction plays in mitochondrial movement, we double stained differentiated PD and CNT cybrid neuronal cells with antibodies to essential components of mtETC complexes I-V. The presence of staining with monoclonal antibodies directed against labile subunits of each complex (CI-NDUFB4, CII-30 kDa, CIII-Core 2 protein, CIV-subunit I, CV-OSCP) indicates that the complex is correctly assembled. [47] By double staining with antibodies to a control subunit (CV α), it was possible to calculate the percentage of cybrid neuronal cells that had intact assembly of complexes I-V (Figure 3). All five complexes were correctly assembled in CNT cybrid neuronal cells (data not shown). PD cybrid neuron lines (PD60, PD61) exhibited an increase mitochondrial movement after LLLT that did not achieve significance. The same lines also exhibited low levels of complexes I and IV assembly as well as reduced assembly in two other complexes (Figure 3). In contrast, the four PD cybrids (PD63, PD65, PD66, PD67) that exhibited a significant response to LLLT contained more cybrid neuronal cells with correctly assembled complexes I and IV (50–100%). In these four PD lines, at least three complexes were correctly assembled which suggests that the mtETC was more functional (Figure 3).

### Oxygen utilization studies

**Figure 4:**
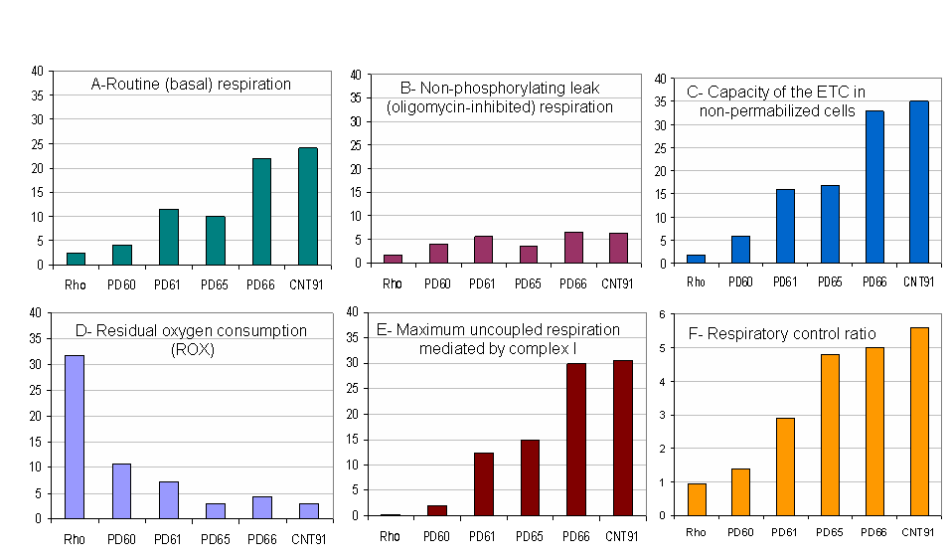
**The graphs in this figure illustrate the levels of routine (basal) respiration (A), nonphosphorylating leak (oligomycin inhibited) respiration (B), capacity of the electron transport chain in nonpermeablized cells (C), residual oxygen consumption or ROX (D), maximum uncoupled respiration mediated by complex I (E) and the respiratory control ratio (F) in Rho0 cells, and selected PD and CNT cybrid neuron lines**. The Y axis in Figures 4A-E is Respiration [pmol O^2^/second/10^-6^ cells]. These data were calculated based on formulas described by Gnaiger. [48]

An Oxygraph 2K (Oroboros) [48] was used to measure changes in oxygen utilization by Rho0 cells that lack a functional mtETC, two lines of PD cybrid neuronal cells (PD60 and PD61) that did not respond significantly to LLLT, two lines of PD cybrid neuronal cells (PD65 and PD66) that did respond significantly to LLLT and a CNT line (CNT91) of cybrid neuronal cells. These data are shown in Figure 4. Rho0 cells, PD60 and PD61 had reduced routine aerobic respiration (Figure 4A), capacity of the mtETC (Figure 4C) and complex I mediated respiration (Figure 4E) compared to CNT91. [see [48] for details] Non-phosphorylating (oligomycin-inhibited) respiration (LEAK, Figure 4B) did not vary greatly among any of the cell lines tested; however the residual oxygen consumption (ROX, Figure 4D) was higher in Rho0, PD60 and PD61 lines compared to PD65, PD66 or CNT91. In contrast CNT91, PD65 and PD66 had higher routine aerobic respiration, mtETC capacity, and complex I mediated respiration (Figures 4A, 4C and 4E) as well as a reduced level of ROX (Figure 4D). The respiratory control ratio (RCR) which reveals the relative efficiency of ETC coupling (Figure 4F) was diminished in Rho0, PD60 and PD61 compared to cybrid lines that responded significantly to LLLT (PD65, PD66 and CNT91). During the two hour period of measurement after LLLT there was no statistically significant change in respiratory chain activity in any of the cybrid lines tested using the Oxygraph-2k (data not shown).

## Discussion

In this study we showed that the velocity of mitochondrial movement by axonal transport was significantly reduced in a human neuronal model of sporadic PD. Other studies have also suggested a role for reduced axonal transport in the pathogenesis of PD. Our data lends additional support for this proposition and for the role that dysfunctional mitochondria play in early PD pathogenesis. We also showed that a single treatment with near-infrared laser light can restore the velocity of mitochondrial movement in PD cybrid neuronal cells to CNT levels. LLLT with near-infrared laser light has been proven safe and effective for the treatment of complex neurological conditions like stroke in humans and rodents. [40,49] Our results provide essential data to support the exploration of LLLT with near-infrared laser light for treatment of neurological conditions like PD.

The mitochondria in PD cybrids exhibit structural and functional changes that could interfere with normal movement of mitochondria by axonal transport or contribute to the differential response of PD cybrid mitochondria to LLLT. These include-1) reduced activity of complexes I and IV, 2) increased levels of oxidized and nitrated proteins, 3) insufficient ATP, 4) a swollen and abnormal morphology, 5) increased levels of modified α-synuclein, and 6) increased free/polymerized tubulin ratio. [24,28,50,51]

A substantial body of evidence supports the proposal that complex I dysfunction plays a critical role in PD pathogenesis. [52] Analysis of the mtETC revealed reduced assembly of complex I in five out of six PD cybrid neuronal cell lines (Figure 3). Previous studies suggest that mtETC subunit expression was also altered in sporadic PD brain. [30] Complex I subunits were also oxidatively damaged and this damage correlated with decreased function in sporadic PD brain. [30] Findings of reduced capacity of the electron transport chain, routine respiration and complex I mediated oxygen utilization (Figure 4) in PD cybrid neuronal cells, complimented our mtETC assembly data. CNT91 cybrid neuronal cells had oxygen utilization rates comparable to fibroblasts, peritoneal mesothelial cells and hematopoetic cells. [48,53,54]

Complex IV is also misassembled in four out of six PD cybrid lines (Figure 3). Reduced complex IV activity and increased levels of nitrated proteins have been detected in PD tissue and in PD cybrid lines[24,27]. Studies have suggested that nitrative damage plays an important role in PD. [55] Both complexes I and IV are targets of NO. [43] Treatment of neuronal cultures with NO reduces the movement of mitochondria and synaptic vesicles; therefore, it is reasonable to propose that nitrative damage to the mtETC contributes to the loss of mitochondrial movement in PD cybrids. [56-58]

While the degree of mtETC misassembly in each PD cybrid line did not correlate with mitochondrial velocity, mtETC misassembly did relate to the response of PD cybrid neuronal cells to LLLT. All the PD cybrid neuronal cells showed increased mitochondrial velocity after LLLT; however the changes in PD60 and PD61 did not consistently achieve significance (Figure 3). Current understanding suggests that near-infrared light is absorbed by photosensitizers such as the oxidized copper centers in complex IV. This results in the excitation and transfer of electrons to oxygen and the generation of low level changes in ROS expression that activates cellular processes through the release of transcription factors and altered gene expression. [41,59,60] Near-infrared light can also reverse the inhibition of complex IV and cellular respiration caused by NO occupation of the oxygen site. [43] Cybrid neuronal cells PD60 and PD61 had the lowest levels of complex I and IV assembly of all our lines (Figure 3). It's not surprising therefore that the PD cybrid lines with the least intact complex I and IV assembly also have a limited response to near-infrared light. LLLT was able to significantly increase mitochondrial movement in other less crippled PD cybrid neuronal cells (Figure 3). LLLT did not improve oxygen utilization in OxyGraph studies of selected PD or CNT lines of cybrid neurons (data not shown). The two hour period selected for this study may not have been sufficient time for changes in mtETC activity to achieve detectable levels. Further studies will focus on longer post-LLLT intervals.

The velocity of mitochondrial movement in CNT cybrid neuronal cells was not altered by LLLT. LLLT also failed to enhance respiratory function in control animals used for a study of rotenone induced mitochondrial optic neuropathy. [35] Rojas et al[35] suggest that mitochondria that are functioning at a maximal rate are unable to be further stimulated by LLLT. On the other hand, if the mtETC is partially inhibited or dysfunctional, LLLT can increase electron flow by stimulating complex IV. [35] Therefore, we were not surprised when the velocity of mitochondrial movement in CNT cybrid neuronal cells did not respond significantly to LLLT in our study.

Reduced ATP levels stemming from mtETC dysfunction could also limit availability of ATP for use by the motors that drive axonal transport. [61] Esteves et al[24] detected reduced levels of ATP in their undifferentiated NT2 PD cybrids. Recent studies have suggested regional increases in ADP can trigger mitochondrial movement. [62] We used the BioVision Research Products ApoSensor ADP/ATP ratio assay kit (Mountain View, CA) to measure the ADP/ATP ratio but could not detect consistently altered levels of ATP or ADP levels (data not shown) in our PD and CNT cybrid neuronal cells after LLLT. Further studies are needed to verify the role reduced ATP or increased ADP play in the reduced movement of mitochondria in PD cybrid neuronal cells.

The mitochondria in valinomycin-treated cerebellar neuronal cells were swollen and they blocked mitochondrial movement. [63] The mitochondria in PD cybrid neurites were not swollen or rounded (Figure 1) so the reduction in movement can not be attributed to this cause. PD cybrids did contain aggregated, oxidized [27] and phosphorylated α-synuclein (data not shown). In transfected neuronal cells, Saha et al [64] showed that mutated and phosphorylated α-synuclein reduced axonal transport. Modified α-synuclein may contribute to the reduction in mitochondrial movement in PD cybrid neurites but it is unlikely that LLLT could alter α-synuclein aggregation, oxidation or phosphorylation in the period of time studied. Aggregates of α-synuclein can appear in PD cybrid neurites and in rotenone-treated SH-SY5Y neuronal cells, cause localized swelling that resembles a Lewy neurite and disrupt movement of all mitochondria distal to the swelling ([2] and data not shown). Lewy neurite-like swellings were uncommon in PD cybrid neurites and did not account for the reduced movement of mitochondria in PD cybrid neuronal cells.

## Conclusion

Speculation that reduced axonal transport plays a role in PD pathogenesis has existed for some time. Our ability to assess the role of axonal transport in PD has improved recently with the development of appropriate models and methods. The outcome of our studies of human cybrid neuronal cells that contain mitochondria derived from CNT and PD volunteers illustrates that axonal transport of mitochondria is significantly reduced in PD cybrid neurites. Reduced axonal transport could underlie the loss of neurites through terminal degeneration and result in the death of dopaminergic neuronal cells in PD. The observation that a single, low level exposure to LLLT can increase the velocity of mitochondrial movement and restore it to CNT levels in PD cybrid neurites is remarkable but consistent with our current understanding of the mechanism of LLLT. In a recent publication, behavioral, metabolic and anatomic measures were improved in a dose-dependent manner in a rat model of rotenone-induced optic neuropathy. [35] LLLT is also currently used to treat a wide range of human conditions that involve injury and inflammation. Recent findings that LLLT can be used to ameliorate the consequences of stroke in phase 2 clinical trials suggest that LLLT with near-infrared light [40] should be explored for the treatment of other neurological diseases such as PD.

## Competing interests

JS and LDT are employees and shareholders in PhotoThera, Inc. UO was an employee of PhotoThera, Inc. during 2006–2007. PAT, KMS and MKB have no competing financial interests.

## Authors' contributions

PAT designed experiments and conducted the axonal transport studies and wrote the manuscript; KMS prepared differentiated cybrid neuronal cells and conducted the oxygraph studies; MKB produced the cybrid lines and conducted the immunocytochemical analysis of mtETC; LDT and JS contributed to the optical and thermal design (dose selection), and reviewed the manuscript; UO contributed to the optical, thermal and experimental design and reviewed the manuscript.

## Supplementary Material

Additional file 1 **PD63 cybrid neuron process**. Time lapse movie of MtRed-labeled mitochondria in a PD63 cybrid neuron process (pass 25) after differentiation with 6 nM staurosporine for 12 days. Click here for file

Additional file 2 **PD63 cybrid neuron process 85 minutes after LLLT with 50 mW of 810 nm light for 40 seconds**. Time lapse movie of MtRed-labeled mitochondria in a PD63 cybrid neuron process (pass 25) after differentiation with 6 nM staurosporine for 12 days. This movie was recorded 85 minutes after LLLT with 50 mW of 810 nm light for 40 seconds. Click here for file

Additional file 3 **CNT68 cybrid neuron process**. Time lapse movie of MtRed-labeled mitochondria in a CNT68 cybrid neuron process (pass 24) after differentiation with 6 nM staurosporine for 12 days. Click here for file

Additional file 4 **CNT68 cybrid neuron process 90 minutes after LLLT with 50 mW of 810 nm light for 40 seconds**. Time lapse movie of MtRed-labeled mitochondria in a CNT68 cybrid neuron process (pass 24) after differentiation with 6 nM staurosporine for 12 days. This movie was recorded 90 minutes after LLLT with 50 mW of 810 nm light for 40 seconds. Click here for file
